# Long-Term Outcome and Comparison of Treatment Modalities of Temporal Bone Paragangliomas

**DOI:** 10.3390/cancers13205083

**Published:** 2021-10-11

**Authors:** Erdem Yildiz, Valerie Dahm, Wolfgang Gstoettner, Karl Rössler, Belinda Bauer, Alexander Wressnegger, Ursula Schwarz-Nemec, Brigitte Gatterbauer, Christian Matula, Christoph Arnoldner

**Affiliations:** 1 Department of Otorhinolaryngology, Head and Neck Surgery, Medical University of Vienna, 1090 Vienna, Austria; erdem.yildiz@meduniwien.ac.at (E.Y.); wolfgang.gstoettner@meduniwien.ac.at (W.G.); n01427032@students.meduniwien.ac.at (B.B.); christoph.arnoldner@meduniwien.ac.at (C.A.); 2 Department of Neurosurgery, Medical University of Vienna, 1090 Vienna, Austria; karl.roessler@meduniwien.ac.at (K.R.); brigitte.gatterbauer@meduniwien.ac.at (B.G.); christian.matula@meduniwien.ac.at (C.M.); 3 Department of Biomedical Imaging and Image-Guided Therapy, Division of Neuroradiology and Musculoskeletal Radiology, Medical University of Vienna, 1090 Vienna, Austria; alexander.wressnegger@meduniwien.ac.at (A.W.); ursula.schwarz-nemec@meduniwien.ac.at (U.S.-N.)

**Keywords:** paraganglioma, temporal bone, skull base, Fisch, Glasscock–Jackson, glomus tympanicum, glomus jugulare

## Abstract

**Simple Summary:**

Temporal bone paragangliomas are rare benign skull base tumors. They are categorized according to Fisch or Glasscock–Jackson classification systems. The complexity of tumor location and extension into neighboring anatomical structures together with multiple treatment alternatives make it difficult to find optimal therapy for patients. In our retrospective study, we evaluated treatment strategies consisting of surgical resection, radiotherapy or radiosurgery and embolization over an extensive long-term follow-up period. We observed that especially small tumors (Fisch A and B) are best treated by surgical resection, and larger temporal bone paragangliomas (Fisch C and D) may be treated with combination therapy. Especially in larger tumors, evaluation in an interdisciplinary board is important.

**Abstract:**

Introduction: Temporal bone paragangliomas are rare tumors with high vascularization and usually benign entity. A variety of modalities, including gross total resection, subtotal resection, conventional or stereotactic radiotherapy including gamma-knife, embolization, and wait-and-scan strategy can be considered. The aim of this study was to compare long-term outcomes of different primary treatment modalities in temporal bone paragangliomas. Materials and Methods: Patients with temporal bone paragangliomas treated between 1976 and 2018 at a tertiary referral center were retrospectively analyzed in this study. Collected patient data of 42 years were analyzed and long-term results including interdisciplinary management were assessed. Patient outcomes were compared within the different therapy modalities according to tumor control rate and complications. Clinical characteristics, radiological imaging, tumor extent and location (according to Fisch classification), symptoms, and follow-up were evaluated and a descriptive analysis for each treatment modality was performed. Tumor recurrence or growth progression and respective cranial nerve function before and after therapy were described. Results: A total of 59 patients were treated with a single or combined treatment modality and clinical follow-up was 7 (13) years (median, interquartile range). Of the included patients 45 (76%) were female and 14 (24%) male (ratio 3:1) with a patient age range from 18 to 83 years. Total resection was performed on 31 patients, while 14 patients underwent subtotal resection. Eleven patients were treated with conventional primary radiotherapy or gamma-knife radiosurgery. Pulsatile tinnitus (*n* = 17, 29%) and hearing impairment (*n* = 16, 27%) were the most common symptoms in our patient group. Permanent lower cranial nerve deficits were observed only in patients with large tumors (Fisch C and D, *n* = 14, 24%). Among the 45 patients who were treated surgically, 88% of patients with Fisch A and B paragangliomas had no recurrent disease, while no tumor growth was perceived in 83% of patients with Fisch C and D paragangliomas. Conclusion: In conclusion, we propose surgery as a treatment option for patients with small tumors, due to a high control rate and less cranial nerve deficits compared to larger tumors. Although patients with Fisch C and D temporal bone paraganglioma can be treated surgically, only subtotal resections are possible in many cases. Additionally, frequent occurrence of cranial nerve deficits in those patients and tumor growth progression in long-term follow-up examinations make a combination of the therapy modalities or a primary radiotherapy more suitable in larger tumors.

## 1. Introduction

Temporal bone paragangliomas (TBPs) are mostly benign neoplasms arising from paraganglion cells along the tympanic plexus and the adventitia of the jugular bulb [1]. Formerly known as “Glomus tympanicum” and “Glomus jugulare”, these paragangliomas are not always categorized uniformly. Tumor staging is often carried out according to Fisch and Mattox [2] or Glasscock–Jackson [3] classification systems. Fisch and Mattox organized both tympanic and jugular paraganglioma into one classification and Sanna et al. [4,5] presented a modified system specifically for tympanic tumors. Fisch A and B TBPs are localized in the tympanic cavity, whereas Fisch C and D TBPs are of jugulotympanic origin. In contrast, the Glasscock–Jackson system divides two classifications each for tympanic and jugular glomus tumors categorized in grades I to IV.

Depending on the TBP growth progression, different cranial nerves (CNs) may be affected. Detailed medical history, neuro-otolaryngological examination and laboratory testing designed to identify an excess of catecholamines is of utmost importance for a correct diagnosis. Familial and multifocal disease and symptoms have to be elicited next to an assessment of a high-resolution computed tomography (CT) and/or magnetic resonance imaging (MRI). Specifically, the salt-and-pepper pattern represents intratumoral flow voids [6]. As intraoperative blood loss may be a serious complication, preoperative diagnostic and treatment planning is essential in evaluating the suitable steps of therapy. Therapeutic options include surgical resection (with or without embolization), conventional radiotherapy, which has evolved into stereotactic gamma-knife radiosurgery (GKRS) in many centers [7], wait-and-scan policy, or palliative embolization [8,9].

The aim of the presented study was to assess patient outcome with different treatment options in a long-term follow-up (FU) time period. Additionally, we sought to compare the outcomes of therapeutic strategies of different sized paragangliomas.

## 2. Materials and Methods

Sixty-eight patients with TBP were treated between 1976 and 2018 at the Vienna General Hospital by the otolaryngology and neurosurgical departments. Nine patients were excluded due to insufficient documentation, resulting in 59 included patients. Two patients were lost to FU, resulting in 57 patients with long-term observation time period of 7 (median; interquartile range, IQR 2–15 years). This was observed in both small and large TBPs (Fisch A/B median FU 7, IQR 9.75 years and Fisch C/D median FU 6, IQR 13 years). FU time was not equally distributed (*p* < 0.0001). Forty-five (76%) female and 14 (24%) male individuals were included with a mean age of 54 years (range 18 to 83 years) at primary therapy. The most common symptoms in patients were hearing loss, pulsatile tinnitus, cranial nerve palsy (CNP) and vertigo. Patient records of different time points prior to therapy and in FU were evaluated.

**Figure 1:**
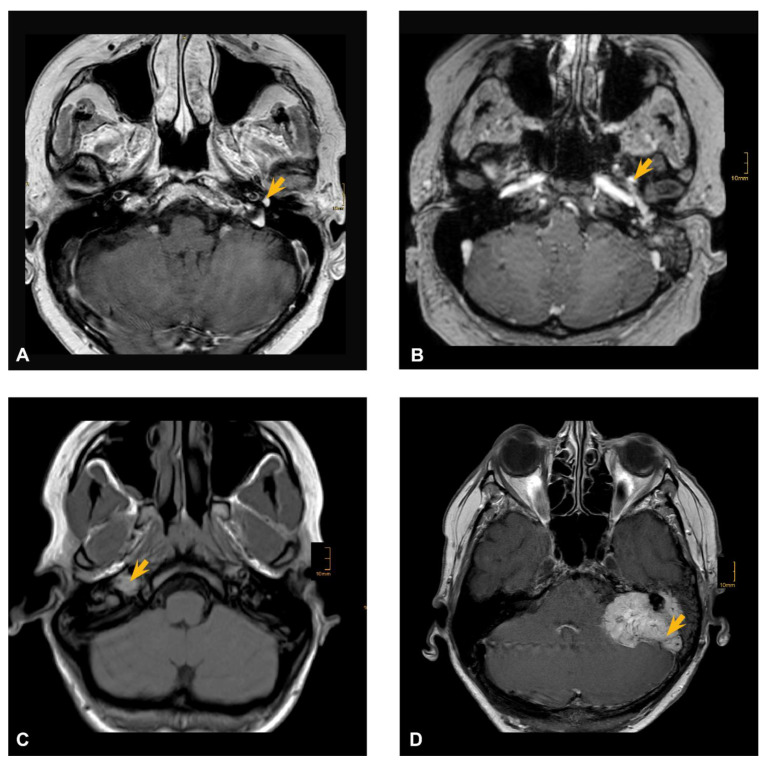
MRI examples of Fisch grades (**A**–**D**) in the respective population. (**A**) cranial MRI scan of Fisch A1 TBP, (**B**) cranial MRI scan of Fisch B3, (**C**) cranial MRI scan of Fisch C1, (**D**) cranial MRI scan of Fisch De2. The yellow arrow indicates tumor location in each radiological scan.

Acquired data included age, sex, date of diagnosis, tumor location, symptoms, CNP, treatment strategy, complications, clinical examination reports, pre- and postoperative CT and MRI imaging and tumor recurrence in FU. Categorization of TBPs according to the Fisch classification system [2] was performed by two experienced neuroradiologists. Figure 1 represents examples of main tumor categories (Fisch A to D) from our patient population. Histological confirmation of paraganglioma was assessed in all patients.

Treatment options consisted of conventional radiotherapy (RT) or gamma-knife radiosurgery (GKRS), subtotal resection (STR) or total resection, single embolization, wait-and-scan strategy, or a combination of the above. In case of primary irradiation therapy, the respective radiation dose in patients with primary conventional RT ranged from 43 to 60 Gy and GKRS from 22 to 28 Gy. Radiation dose in all adjuvant RT cases ranged between 48 to 54 Gy within the conventional RT group and 26 to 30 Gy in case of GKRS.

Patients were retrospectively assigned into groups of each treatment modality and Fisch grade. CNP before and after treatment, as well as in the FU examination were compared. Tumor recurrence and tumor growth progression was described over time after primary treatment. New onset or improvement of CNP was described. Pulsatile tinnitus, hearing impairment and vertigo over time were quantitatively analyzed. Further, embolized patients were observed and compared to non-embolized patients with respect to treatment complications, which consisted of bleeding and CNP.

Descriptive statistical analysis was performed with SPSS Statistics for Mac, Version 23.0 (IBM, Armonk, NY, USA) and Figure illustrations were performed with GraphPad Prism version 9.0.0 for macOS, (GraphPad Software, La Jolla, CA, USA). Shapirow–Wilk test was performed for analysis of normal distribution within the study group. A probability value of *p* < 0.05 was considered statistically significant.

## 3. Results

### 3.1. Study Cohort

**Figure 2:**
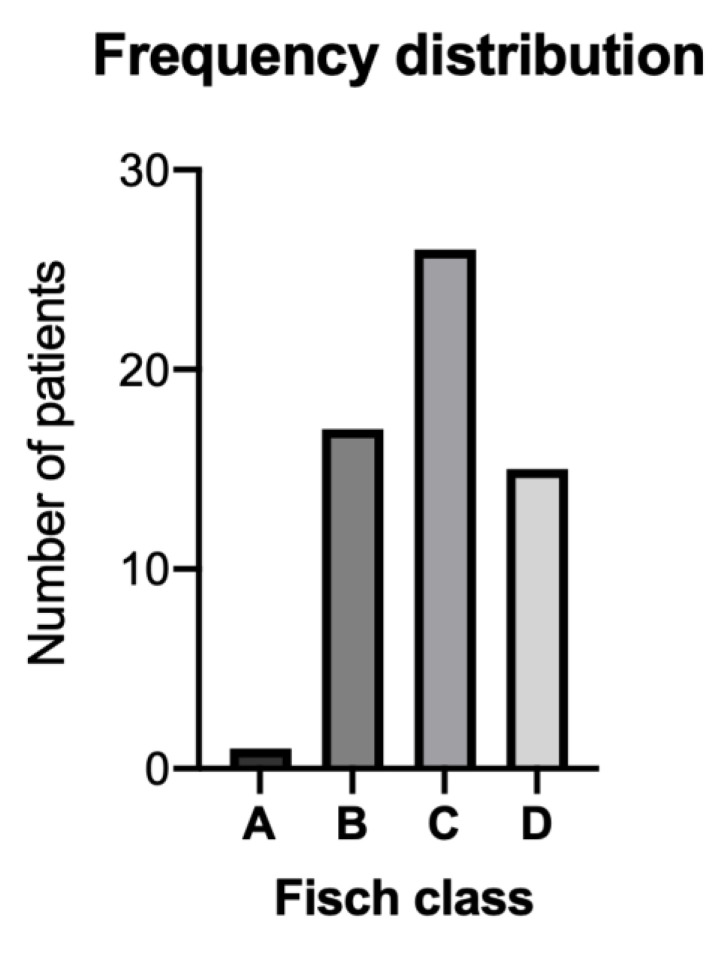
Number of patients clustered according to Fisch classification. **A** = 1, **B** = 17, **C** = 26, and **D** = 15 patients.

In the observed study period, we collected data from 59 patients with TBPs. Within our patient cohort, there was one (2%) Fisch A tumor, 17 (29%) Fisch B, 26 (44%) Fisch C and 15 (25%) Fisch D tumors. Figure 2 shows the patient distribution according to Fisch grades. In 19 (32%) patients, the Fisch subgroup was unknown due to inaccessible archived imaging data. In the remaining cases, majority of tumors were C1 (*n* = 12, 30%) followed by B1 (*n* = 10, 25%) and De1 (*n* = 9, 22.5%). Further, our study cohort includes three B2 tumors and each patient in the subgroups A1, B3, C2, C3, De2, and Di1.

A total of 45 (76%) patients were treated surgically. Eleven patients (19%) underwent either primary conventional RT (*n* = 6) or GKRS (*n* = 5). Observation strategy was selected as “therapy” in two patients due to comorbidities and an extensive tumor (Fisch D) or due to patients’ choice (Fisch B1). One patient (class De1 tumor) underwent embolization but rejected further surgical resection.

### 3.2. Treatment

#### 3.2.1. Surgery

**Figure 3:**
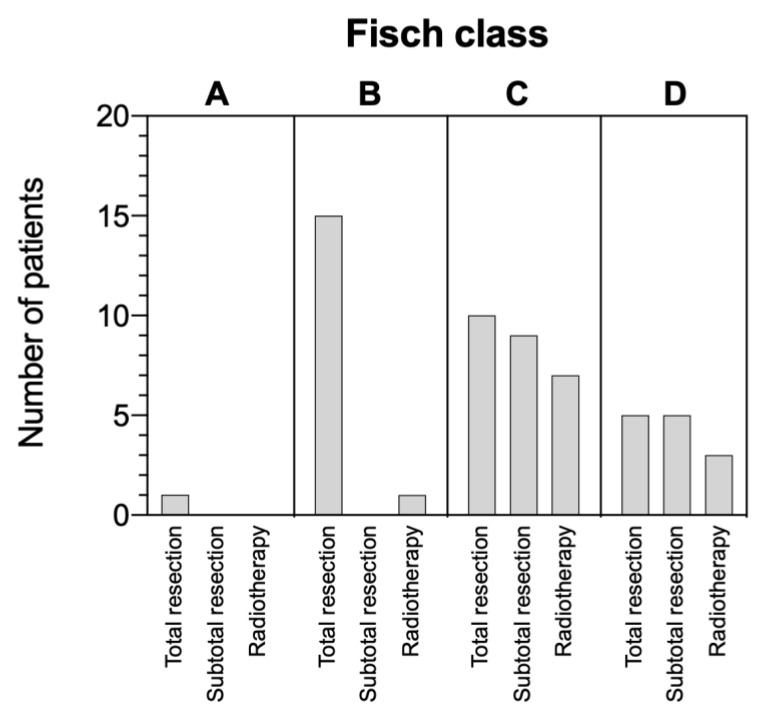
Frequency distribution of different primary therapies in all observed patients. Column **A** represents patients with a paraganglioma classified as Fisch A TBPs. Respectively, column **B** represents Fisch B, column **C** represents Fisch C and column **D** represents Fisch D TBPs. Radiotherapy includes conventional radiotherapy or gamma-knife radiosurgery.

Figure 3 illustrates different therapy modalities and their application on TBPs within our study cohort. Total resection of TBPs was achieved in 31 (69%) patients including all Fisch grades, whereas a subtotal resection was carried out in 14 patients (31%, including only Fisch C and D). All surgically treated Fisch A and B tumors were totally resected (*n* = 16). Within this group, five patients received preoperative embolization (all among Fisch B tumors). None of these patients received further treatment.

Fisch C tumors were completely resected in ten (38%) cases, four with prior embolization. Of those patients, two received adjuvant conventional RT (48 and 50 Gy). All nine (35%) subtotally resected Fisch C TBPs were treated with embolization prior to surgery and an adjuvant RT was performed in seven cases (five conventional RT and two GKRS).

Total resection of Fisch D paragangliomas was possible in five patients. Four of those received a preoperative embolization and one patient underwent adjuvant conventional RT. STR was conducted in five patients with four patients undergoing prior embolization. All five received adjuvant RT (four conventional RT and one GKRS).

#### 3.2.2. Radiotherapy/Radiosurgery

The choice of all primary RTs or radiosurgery was based mostly on large tumor size (Fisch B, *n* = 1, Fisch C and D, *n* = 10). Seven (64%) patients with Fisch C TBP underwent primary RT (4 conventional RT and 3 GKRS). The other cases were three (27%) Fisch D (2 primary RT, 1 GKRS) and one (10%) Fisch B TBP (GKRS).

Various reasons led to the choice of radiotherapy/radiosurgery. In two cases (Fisch B and Fisch D), RT was advised due to advanced patient age. Three Fisch C tumors were treated with RT because of severe pretherapeutic symptoms or cardiac insufficiency which led to contraindication of surgery. In one patient (Fisch D), the tumor covered large parts of the carotid artery and a surgical resection was not advised by our multidisciplinary team.

#### 3.2.3. Treatment Alternatives

A 40-year-old patient with a Fisch De1 TBP received primary embolization therapy. The originally planned surgical resection was rejected by the patient. Due to further tumor growth in FU, this patient underwent multiple radiation therapies instead. In two further cases (Fisch B1 and D), a patient observational strategy was performed (i.e., wait-and-scan). The patient suffering from Fisch B1 TBP denied treatment due to the risk of hearing loss. The other patient with a Fisch D TBP (83 years) declined the recommended radiation therapy. In both cases, a preoperative biopsy confirmed paraganglioma diagnosis.

### 3.3. Pre- and Post-Therapeutic Cranial Nerve Palsy

Among the 59 included patients, eleven (19%) presented with a CNP at tumor diagnosis. This affected only patients with Fisch C and D TBPs. None of the Fisch A or B TBP showed CNPs, neither at time of diagnosis nor in FU. In eight (14%) patients, CN status was unknown at the time of tumor diagnosis and therefore was not included in further calculations. Overall, a CNP was reported in three patients with Fisch C and D TBPs, which were treated surgically. None of the patients treated with radiotherapy or radiosurgery experienced a CNP one year after initial treatment.

Three patients with Fisch C TBP had a CNP at tumor diagnosis. The number of CNP affected patients with Fisch C TBPs rose temporarily to seven but declined to four in the FU examination after one year. At the time of last FU, a permanent CNP was reported in one patient. The affected patient had a C1 TBP and developed a facial palsy shortly after embolization and prior to surgery.

Referring to Fisch D TBP, we observed eight affected patients prior to therapy. This number increased to nine after treatment and further increased up to eleven patients with CNP during the follow-up time period (up to one year). None of the initial eight affected patients experienced any additional paresis. However, two patients obtained a new CNP. Among them, one patient with De1 TBP experienced a permanent facial palsy after surgical treatment. The second patient with Fisch D TBP (subgroup unknown) experienced multiple CNPs (CN VII, X and XI) one year after initial therapy due to tumor growth progression. Details of CNPs in patients with Fisch C and Fisch D paraganglioma are listed in Table 1.

CNs affected by TBPs included VI, VII, X, XI, or XII. These were observed as more than a single CNP. Four patients experienced multiple CNPs prior to therapy. All of them had exceptionally large tumors (two Fisch De1, one Fisch De2 and one Fisch Di2), but showed no additional CNP one year after surgical intervention. In one patient with De1 TBP, the hypoglossal palsy existing prior to therapy reversed within one year post surgery and the patient recovered from it. Descriptive statistics of the observed patient group is included in Table 2. We observed a significantly lower occurrence of CNP in small TBPs (Fisch A and B) before treatment (*p* = 0.0148) and in long-term FU (0.0045).

Of all 59 patients, the majority (*n* = 29; 49%) showed pulsatile tinnitus prior to intervention. This symptom reversed in the surgical and RT/radiosurgery treatment groups in FU examinations. The second most common reported symptom was hearing impairment. A subjective hearing impairment was observed in 27 (46%) patients prior to therapy, but further analysis of this symptom was not assessed after. Subjective vertigo was specified in nine patients but disappeared in four cases at the one year FU examination.

### 3.4. Complications

**Figure 4:**
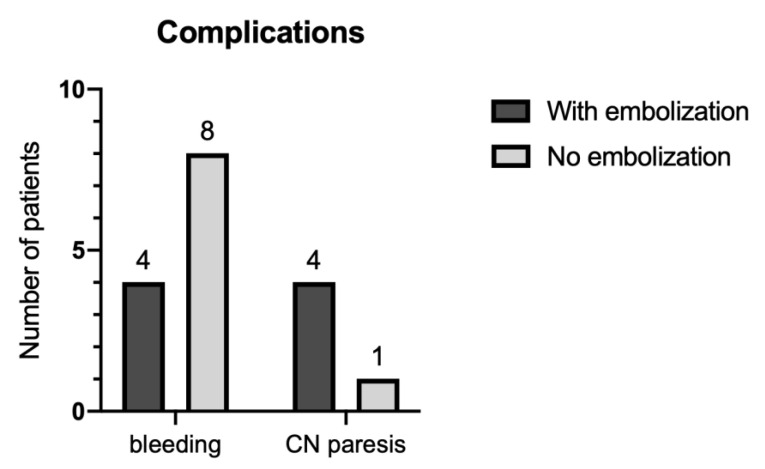
Patients with complications (bleeding and CN paresis) divided into embolized and non-embolized groups. CN cranial nerve.

Perioperative hemorrhage complications were observed in twelve patients (27%) across all Fisch grades. Intraoperative bleeding was documented in eleven patients whereas one patient with Fisch De2 TBP had to undergo revision surgery due to postoperative bleeding. Patients with preoperative embolization had less perioperative bleeding compared to non-embolized patients (See Figure 4). However, the number of CN paresis in embolized patients exceeded the number of non-embolized patients. These were reversible in all cases except one.

### 3.5. Tumor Recurrence and Progression

**Figure 5:**
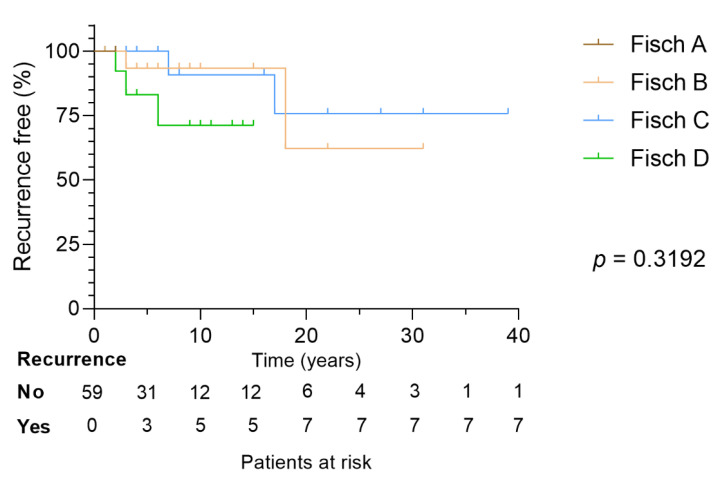
Kaplan–Meier curve and log-rank test of recurrence free patients over the long-term follow-up period. No significant differences between Fisch A to D TBPs were observed (*p* = 0.3192). TBP—temporal bone paraganglioma.

In 57 (97%) patients, an FU examination of at least one year after primary treatment was carried out. Of these, 16 (28%) patients experienced a TBP recurrence or a tumor growth progression. While 14 (88%) of those patients were originally treated surgically (including complete and partial resection), one patient with Fisch De1 TBP had undergone conventional RT (43 Gy), and another patient with Fisch De1 TBP received initial embolization and further conventional RT (50 Gy) but underwent surgery three years after RT due to tumor progression. As indicated in Table 2, there was no significant difference in tumor recurrence when tumor size (according to Fisch, *p* = 0.9056) or treatment alternative were compared (surgery vs. RT/GKRS, *p* = 0.1620). A log-rank test for TBP recurrence within the long-term FU revealed no significant differences between all different Fisch tumors in our patient group (*p* = 0.3192, Figure 5).

Surgical resection was the primary treatment option in 45 of 57 (79%) patients. Of these, eight (18%) showed TBP recurrence at one to 18 years after initial tumor resection. Among them, one patient with Fisch C3 TBP did not need further treatment during 19 years of FU, although a tumor recurrence was observed. Observational strategy was chosen in this patient up to the time point of our retrospective analysis. Two patients (Fisch B1 and De1 TBP) experienced a tumor recurrence 18 and two years after gross total resection and were treated with conventional radiotherapy (54 and 50 Gy) and tumor was kept under control 23 and 26 years into the second treatment. A third patient with C3 TBP developed a tumor recurrence 17 years after complete resection, but no further tumor progression has been observed. All remaining patients with complete tumor resection (one Fisch B, one Fisch C and two Fisch D TBP) experienced multiple tumor recurrences and were treated surgically (*n* = 2), with GKRS (*n* = 1) or only with embolization (*n* = 1). Within those patients tumors remain under control 14 to 34 years after the latest treatment.

Residual tumor growth was observed in six (13%) patients one to 13 years after initial STR. While all of them had a Fisch C TBP, four received adjuvant RT. One patient with Fisch C1 TBP needed no further therapy 16 years into FU. Another patient with Fisch C TBP underwent re-operation after initial STR due to tumor regrowth two years into initial surgery. Tumor progression was observed in this patient 20 years after the second operation. After a third surgical resection, tumor growth remained under control eleven years after the last treatment. Remaining patients with Fisch C TBPs underwent RT as a second treatment (one GKRS with 28 Gy and three conventional RT with either 50Gy or 54Gy) one to 13 years after initial STR and tumors remain under control in further FU examinations.

In 88% of all Fisch A and B tumors with surgical resection, a successful tumor control was achieved (14 of 16). This number declined in larger tumors as 83% (24 of 29) of surgically resected Fisch C and D TBP had no recurrence or residual growth of tumor, which needed additional treatment. Among eleven patients primarily treated with either RT or GKRS, two were lost to FU (both GKRS). Within the remaining nine patients, only one (11%) tumor progression was observed.

## 4. Discussion

In our retrospective study, we present results of all primary therapy modalities on 59 patients who suffered from TBP. We present a long-term FU in 57 of those patients within the observed time period of 42 years. Since there is no gold standard for the treatment of TBP, the evaluation of each individual patient record is essential. TBPs are located at the lateral skull base and pose challenging obstacles when it comes to the correct choice of each patient treatment modality. Due to the relative proximity to important neurovascular structures, a tumor growth can lead to lower CN deficits. Traditionally, the definitive treatment of TBPs has been a surgical resection [10,11]. However, the complexity of these tumors regarding origin, extent and variability in clinical presentation inevitably leads to a necessary critical discussion of treatment modality. For this reason, different treatment strategies might be chosen in various centers (see Table 3). Most authors recommend an initial wait-and-scan strategy [12,13,14]. Kuenzel et al. suggest primary surgical procedure as the treatment of choice particularly in young patients with unilateral tumors and CN paresis [13]. Cheesman et al. conclude that in patients with normal CN function and normal hearing function, a subtotal resection may be sufficient as treatment [15], and Cosetti et al. advocate conservative surgical excision and vigilant long-term monitoring in elderly patients with TBP after studying their patient group with a mean age of 74.5 years [16]. Similar to Nicoli et al. [17], our strategy consists of primary radical surgery whenever complete tumor resection is possible. Further, we perform a subtotal resection or a combined treatment (surgery and RT or GKRS) when radical resection remains impossible without substantial comorbidities. This is also the case when tumor compression leads to severe symptoms which affect the quality of life of patients.

In a previous manuscript, we presented the results of 37 treated TBP patients [18]. The present work demonstrates a larger patient population with a longer FU time period and updated treatment modalities. Our results confirm the higher incidence of TBPs in females with the sex ratio being approximately 3:1. Similar to previous studies, we report that the majority of Fisch A and B paragangliomas underwent a total resection, while around 34% of Fisch C and D tumors underwent subtotal resection. Additionally, primary RT or GKRS as a treatment modality was chosen for larger tumors (5 GKRS, 6 RT) with the exception of one Fisch B tumor treated with GKRS. A local control rate of 88% was achieved in surgically treated Fisch A and B tumors and 83% in Fisch C and D tumors. This fact confirms the viewpoint that radical tumor excision may still be recommended in TBPs [13,20]. Primary surgery seems especially appropriate in Fisch A and B TBPs as those were totally resectable in all patients without functional deficiencies of CNs. Especially when considering CNPs, treatment of larger TBPs (Fisch C and D TBP) remains challenging. As indicated in our results (Table 1), the number of CNPs increase with the tumor size. This was the case prior to TBP treatment and after intervention. The appearance of complications, especially CN deficits of each therapy modality, must be analyzed carefully. Next to postoperative additional CN deficits due to embolization prior to surgery in one patient with Fisch C TBP, we report one patient with De1 TBP, who acquired facial palsy after surgical intervention. Further, we observed two surgically treated patients (Fisch D TBPs) with new CNPs after therapy. However, those CN deficits were observed in FU examinations one year after initial treatment due to tumor progression and were not a surgical complication. As large tumors can often only be resected subtotally in order to spare neighboring essential anatomical structures, a combined therapy with RT including a narrow radiological FU seems to be a suitable option. Nevertheless, we do not know whether a primary radiotherapy might be a sufficient alternative to a combined therapy in large tumors as the observed patient cohort was treated primarily with a surgical or combined therapy and RT/GKRS alone was sparse. If this is applicable, a tumor reduction before RT could be obsolete or only favored under rare circumstances.

Although only one of nine patients treated with RT alone showed a recurrent (or newly onset) tumor growth, a comparison of surgery vs. radiotherapy/radiosurgery was not possible due to a low number of cases in this subgroup. For this reason, we cannot make a statement regarding the recommendation of radiotherapy/radiosurgery alone. A recent meta-analysis conducted by Sahyouni et al. analyzes TBPs treated with radiotherapy [21]. There, a tumor control rate of 94.5% represented by all Fisch grades were pooled. Another meta-analysis performed by Guss et al. reported a 97% tumor control rate in patients with TBP, who were treated either with GK, linear accelerator-based radiosurgery or Cyberknife [22]. Of these patients, unchanged or improved clinical status were reported in 95% of patients. These data suggest consideration of primary radiotherapy over surgical therapy in cases of large tumors. However, we must consider that relatively low FU times observed (mean FU >36 months in 10 included studies) and that studies lack of long-term results, especially GKRS, which is a relatively new treatment option (established in our institution since 1992) when compared to surgery. Further FU data of patients with primary RT are required to conclude the efficacy and long-term results. Nevertheless, Ivan et al. report in their extensive meta-analysis a tumor control rate of 95% in patients treated with stereotactic radiosurgery alone. This group consisted mainly of Fisch C and D TBPs (96%) and were observed for up to 6 ± 0.4 years (mean ± SEM) [23].

Jansen et al. presented, in a recent retrospective multicentric cohort study, the outcome of TBP of Fisch class C and D [24]. They found the highest local control rate (100%, *n* = 19) in TBP when a combined therapy, consisting of tumor debulking and postoperative RT, was performed. The same group reported, in a recent systematic review of 18 publications, excellent local control post-surgery in Fisch A and B tumors. Additionally, they reported a tumor control rate (oder tumor control rates ohnw “a”) of 84% in C1–4 tumors post-radiotherapy, while tumor control was 80–95% after surgery within the same Fisch class group [20]. It can be assumed that these varying numbers of tumor control rates in different treatment modalities reflect the problem of study limitations within the included meta-analyses. Due to the rare entity of paragangliomas, it is difficult to adjust selection criteria to include as many studies as possible.

Certainly, the development of intraoperative neuromonitoring techniques in recent years contributed to the fact that CN deficits can be avoided in more patients due to surveillance of nerve function during tumor resection. Another interesting factor we observed is the fact that in eight patients treated without preoperative embolization, a bleeding complication was documented, while only one patient had a CN paresis (Figure 4). Conversely, only four patients with embolization suffered intraoperative bleeding complication, all with a postoperative CN deficit.

### 4.1. Study Limitations

Our study includes limitations that are common in every retrospective data collection. Despite the rarity of TBPs, we observed a relatively large study cohort. However, patients within our study group received various treatment modalities, thus resulting in smaller group sizes for each modality. This is especially the case in patients primarily treated with radiotherapy/radiosurgery. Although we report an observed time period of over four decades, a long-term FU was not documented in two patients. In spite of these limitations, we could observe patients with tumor recurrences at advanced FU time points. Moreover, our study presents a relatively large group of surgically treated patients whose outcome bring a significant contribution in the choice of initial therapy for paragangliomas.

Finally, the various treatment options in Fisch C and D TBP can be re-evaluated when further data are available in future studies. With reference to our results, we can conclude that surgery in these tumor grades is a valuable option and must be considered in case discussions. However, irradiation therapy in larger tumors may be preferred. Considering Fisch A and B TBPs, our data confirm that the best treatment modality remains the surgical resection.

## 5. Conclusions

In conclusion, we propose surgery as a treatment option for patients with small tumors due to a high control rate and less CN deficits compared to larger tumors. Although patients with Fisch C and D temporal bone paraganglioma can be treated surgically, only subtotal resections are possible in many cases. Additionally, frequent occurrence of CN deficits in this patient group and tumor progression in long-term follow-up examinations make a combination of treatment modalities or a primary GKRS/radiotherapy more suitable in larger tumors. In the setting of surgery of large tumors, the option of embolization prior to therapy should be discussed in an interdisciplinary setting to evaluate the risk of CN paresis versus intraoperative bleeding complication for the individual patient.

## Figures and Tables

**Table 1:** Frequency of cranial nerve deficiencies categorized by treatment modality.

Time Point	No	Fisch C		No	Fisch D		Total
At diagnosis	3	Surgery	RT	8	Surgery	RT	11
Directly after treatment	4	3	1	1	1	0	5
12 months FU	1	1	0	2	2	0	3

Columns “Surgery” and “RT” represent additional paresis after treatment modality. Columns “No” represent the number of affected patients. Rows indicate number of patients with pre-existing or new CNP at each time point. For the sake of overview, column “Total” represents exclusively new acquired paresis at time points “Directly after treatment” and “12 months FU”. CNP, cranial nerve palsy; FU, follow-up; RT, radiotherapy.

**Table 2:** Descriptive statistics of patient population with variables gender, age, Fisch class and treatment alternative.

Variables	Total				
	*n* (%)				
**Sex**	59 (100)				
Female	45 (76)				
Male	14 (24)				
Age (median ± SD)	56 ± 17.4	Pretreatment CNP		CNP FU	
Classification		*n* (%)	*p* value	*n* (%)	*p* value
Fisch A/B	18 (31)	0	0.0148 ^a^	0	0.0045 ^a^
Fisch C/D	41 (69)	11		14	
Treatment					
Surgery	45 (76)	9 (15)	0.8918 ^a^	12 (20)	0.5602 ^a^
RT/GKRS	11 (19)	2 (3)		2 (3)	
Embolization	1 (2)	0	-	0	-
Wait and See	2 (3)	0	-	0	-
		TBP Recurrence in FU			
			
		*n* (%)	*p* value		
Classification					
Fisch A/B	18 (31)	2 (11)	0.9056 ^a^		
Fisch C/D	41 (69)	5 (12)			
Treatment					
Surgery	45 (76)	7(16)	0.1620 ^a^		
RT/GKRS	11 (19)	0			
Other	3 (5)	-			

CNP was observed before treatment and in FU. TBP recurrence was observed at FU time point. ^a^ Chi-square test; CNP, cranial nerve palsy; FU, follow-up; RT, radiotherapy; TBP, temporal bone paraganglioma; GKRS, gamma-knife radiosurgery.

**Table 3:** Comparison of treatment protocols and results in Fisch A to D TBPs among different centers.

Study	*n*—Patients	FU Time Mean (Range)	Treatment	N—New CNP at FU	N—TBP Recurrence
Moe et al. 1999 [10]	132 (83 with FU)	2.1 (2–11) years	Surgery	100 (76%) *	1 (1.2%)
Gstoettner et al. 1999 [18]	37	8.6 (2–15) years	Surgery (*n* = 28)	4 (10.8%)	0
			RT (*n* = 9)	0	0
Cosetti et al. 2008 [16]	12	7.8 (2–33) years	Surgery (*n* = 7)	0	1 (8.3%)
			RT/Wait & See (*n* = 5)	0	0
Kuenzel et al. 2012 [13]	45	3.9 years	Surgery (*n* = 29)	7 (3%)	Surgery 2 (6.9%)
			RT/GKRS (*n* = 12)	2 (16.7%)	0
			Wait & See (*n* = 4)	-	-
Duzlu et al. 2016 [19]	34	2.15 (0.3–9) years	Surgery	5 (14.7%)	0
Nicoli et al. 2017 [17]	36	6.1 (0.5–37) years	Surgery (*n* = 34)	4 (11.8)	5 (2.8%)
			RT (*n* = 2)	1 (50%)	0
This study	59	10.3 (1–39) years/ Median 7 (IQR 2–15) years	Surgery (*n* = 45)	3 (6.7%)	7 (15.6%)
			RT/GKRS (*n* = 11)	0	-
			Wait & See (*n* = 2)	0	-
			Embolization (*n* = 1)	0	-

The study marked with asterisk (*) includes patients with preoperative CNPs. Extraction of patients exclusively acquired CNPs was not feasible. CNP, cranial nerve palsy; FU, follow-up; N, Number of; RT, radiotherapy.

## Data Availability

The data presented in this study are available in this article.

## Abbreviations

CT	Computed tomography
CN	Cranial nerve
CNP	Cranial nerve palsy
FU	Follow-up
GK	Gamma-knife
GKRS	Gamma-knife radiosurgery
IQR	Interquartile range
MRI	Magnetic resonance imaging
RT	Radiotherapy
SEM	Standard error of the mean
STR	Subtotal resection
TBP	Temporal bone paraganglioma

## References

[1] Weissman J.L., Hirsch B.E. (1998). Beyond the promontory: The multifocal origin of glomus tympanicum tumors. Am. J. Neuroradiol..

[2] Fisch U., Pillsbury H.C. (1979). Infratemporal fossa approach to lesions in the temporal bone and base of the skull. Arch. Otolaryngol..

[3] Jackson C.G., Welling D.B., Chironis P., Glasscock M.E., Woods C.I. (1989). Glomus tympanicum tumors: Contemporary concepts in conservation surgery. Laryngoscope.

[4] Sanna M., Fois P., Pasanisi E., Russo A., Bacciu A. (2010). Middle ear and mastoid glomus tumors (glomus tympanicum): An algorithm for the surgical management. Auris. Nasus. Larynx..

[5] Shin S.H., Sivalingam S., De Donato G., Falcioni M., Piazza P., Sanna M. (2012). Vertebral artery involvement by tympanojugular paragangliomas: Management and outcomes with a proposed addition to the fisch classification. Audiol. Neurootol..

[6] Sweeney A.D., Carlson M.L., Wanna G.B., Bennett M.L. (2015). Glomus tympanicum tumors. Otolaryngol. Clin. North. Am..

[7] Foote R.L., Pollock B.E., Gorman D.A., Schomberg P.J., Stafford S.L., Link M.J., Kline R.W., Strome S.E., Kasperbauer J.L., Olsen K.D. (2002). Glomus jugulare tumor: Tumor control and complications after stereotactic radiosurgery. Head Neck..

[8] Persky M.S., Setton A., Niimi Y., Hartman J., Frank D., Berenstein A. (2002). Combined endovascular and surgical treatment of head and neck paragangliomas--a team approach. Head Neck..

[9] Boedeker C.C., Ridder G.J., Schipper J. (2005). Paragangliomas of the head and neck: Diagnosis and treatment. Fam. Cancer.

[10] Moe K.S., Li D., Linder T.E., Schmid S., Fisch U. (1999). An update on the surgical treatment of temporal bone paraganglioma. Skull Base Surg..

[11] Suarez C., Rodrigo J.P., Bodeker C.C., Llorente J.L., Silver C.E., Jansen J.C., Takes R.P., Strojan P., Pellitteri P.K., Rinaldo A. (2013). Jugular and vagal paragangliomas: Systematic study of management with surgery and radiotherapy. Head Neck..

[12] Carlson M.L., Sweeney A.D., Wanna G.B., Netterville J.L., Haynes D.S. (2015). Natural history of glomus jugulare: A review of 16 tumors managed with primary observation. Otolaryngol. Head Neck Surg..

[13] Kunzel J., Iro H., Hornung J., Koch M., Brase C., Klautke G., Zenk J. (2012). Function-preserving therapy for jugulotympanic paragangliomas: A retrospective analysis from 2000 to 2010. Laryngoscope.

[14] Moore M.G., Netterville J.L., Mendenhall W.M., Isaacson B., Nussenbaum B. (2016). Head and Neck Paragangliomas: An Update on Evaluation and Management. Otolaryngol. Head Neck Surg..

[15] Cheesman A.D., Kelly A.M. (2009). Rehabilitation after treatment for jugular foramen lesions. Skull Base.

[16] Cosetti M., Linstrom C., Alexiades G., Tessema B., Parisier S. (2008). Glomus tumors in patients of advanced age: A conservative approach. Laryngoscope.

[17] Nicoli T.K., Sinkkonen S.T., Anttila T., Makitie A., Jero J. (2017). Jugulotympanic paragangliomas in southern Finland: A 40-year experience suggests individualized surgical management. Eur. Arch. Otorhinolaryngol..

[18] Gstoettner W., Matula C., Hamzavi J., Kornfehl J., Czerny C. (1999). Long-term results of different treatment modalities in 37 patients with glomus jugulare tumors. Eur. Arch. Otorhinolaryngol..

[19] Duzlu M., Tutar H., Karamert R., Karaloğlu F., Şahin M.M., Göcek M., Uğur M.B., Göksu N. (2016). Temporal bone paragangliomas: 15 years experience. Braz. J. Otorhinolaryngol..

[20] Jansen T.T.G., Timmers H., Marres H.A.M., Kaanders J., Kunst H.P.M. (2018). Results of a systematic literature review of treatment modalities for jugulotympanic paraganglioma, stratified per Fisch class. Clin. Otolaryngol..

[21] Sahyouni R., Mahboubi H., Moshtaghi O., Goshtasbi K., Sahyouni S., Lin H.W., Djalilianet H.R. (2018). Radiosurgery of Glomus Tumors of Temporal Bone: A Meta-analysis. Otol. Neurotol..

[22] Guss Z.D., Batra S., Limb C.J., Li G., Sughrue M.E., Redmond K., Rigamonti D., Parsa A.T., Chang S., Kleinberg L. (2011). Radiosurgery of glomus jugulare tumors: A meta-analysis. Int. J. Radiat. Oncol. Biol. Phys..

[23] Ivan M.E., Sughrue M.E., Clark A.J., Kane A.J., Aranda D., Barani I.J., Parsa A.T. (2011). A meta-analysis of tumor control rates and treatment-related morbidity for patients with glomus jugulare tumors. J. Neurosurg..

[24] Jansen T.T.G., Kaanders J., Beute G.N. (2018). Timmers, H.; Marres, H.A.M.; Kunst, H.P.M. Surgery, radiotherapy or a combined modality for jugulotympanic paraganglioma of Fisch class C and D. Clin. Otolaryngol..

